# MRI and CT findings of a primary malignant fibrous hystiocitoma presenting as a huge glissonian mass; imaging findings with surgical and histological correlations

**DOI:** 10.1259/bjrcr.20180055

**Published:** 2018-08-02

**Authors:** Federica Romano, Michele Altiero, Ettore Laccetti, Fulvio Calise, Raffaele D’Avino, Giulio Benincasa, Mariano Scaglione

**Affiliations:** 1 Department of Radiology, Pineta Grande Hospital, Castel Volturno, Italy; 2 Department of Surgery, Pineta Grande Hospital, Castel Volturno, Italy; 3 Department of Pathology, Pineta Grande Hospital, Castel Volturno, Italy

## Abstract

The present case report describes imaging findings (CT and MRI features) of a primary malignant fibrous hystiocitoma, presenting as a dual stage lesion, completely exophytic along liver surface with surgical and histological correlations. Imaging characteristics suggested the nature of the lesion (mesenchymal) and the behavior (expansile growth pattern) which addressed surgeons to a conservative excision.

## Introduction

We present a case of primary malignant fibrous hystiocitoma (MFH) arising from liver glisson capsule, surgically confirmed.

To the best of our knowledge, this is the first reported case that shows MFH arising from glisson capsule, with a growth pattern completely exophytic along liver surface.

Imaging characteristics on CT and MRI suggested the encapsulated mesenchymal glissonian nature of the tumor, addressing the therapeutical conservative management with surgical excission of the mass. The resected specimen revealed a malignant fibrous hystiocitoma of 9 × 7 × 4 cm with a double component, as a dual-stage lesion.

## Case presentation

A 70-year-old patient was admitted to our Institute for further examinations, after incidental ultrasound finding of a huge lesion in gallbladder fossa of uncertain origin.

Patient was asymptomatic, laboratory tests as hepatitis B surface antigen and antibodies for the hepatitis C virus were negative, alpha-fetoprotein was within normal limits.

## Investigations

Patient underwent CT examination with triphasic study, and then underwent MRI examination after GD-DTPA e.v. administration.

**Figure 1.**
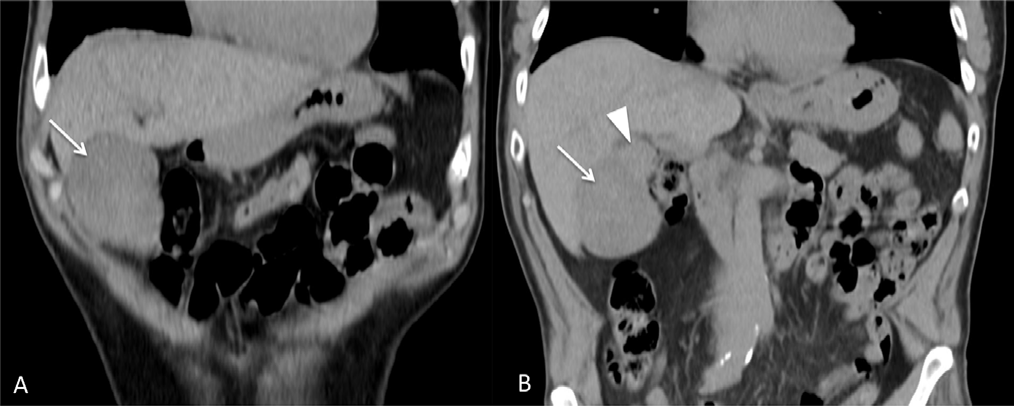
Coronal MPR precontrast CT images show a huge hypodense lesion along liver surface (long arrow) with a cranial round isodense nodule (arrowhead).

**Figure 2.**
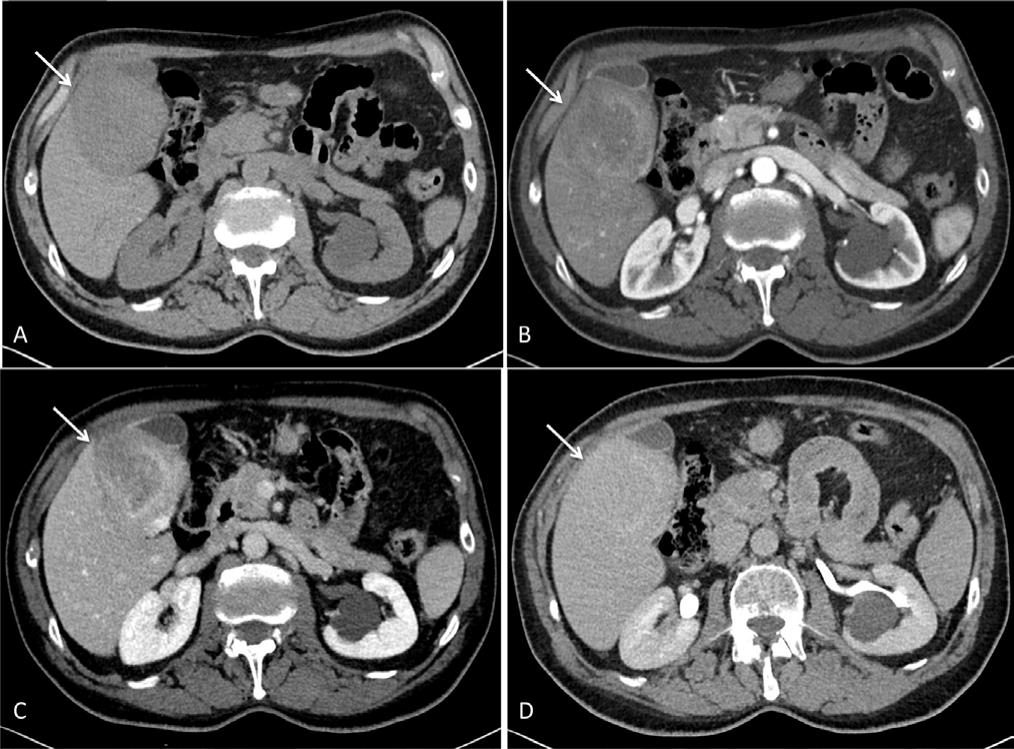
Precontrast CT image (A) shows a weak heterogeneous hypodense mass along liver and gallbladder surface (arrow) contrast-enhanced CT at the arterial phase shows peripheral enhancement (B) with more progressive irregular centripetal fill-in (C) and therefore iso-hyper-attenuation to liver parenchyma (D).

**Figure 3.**
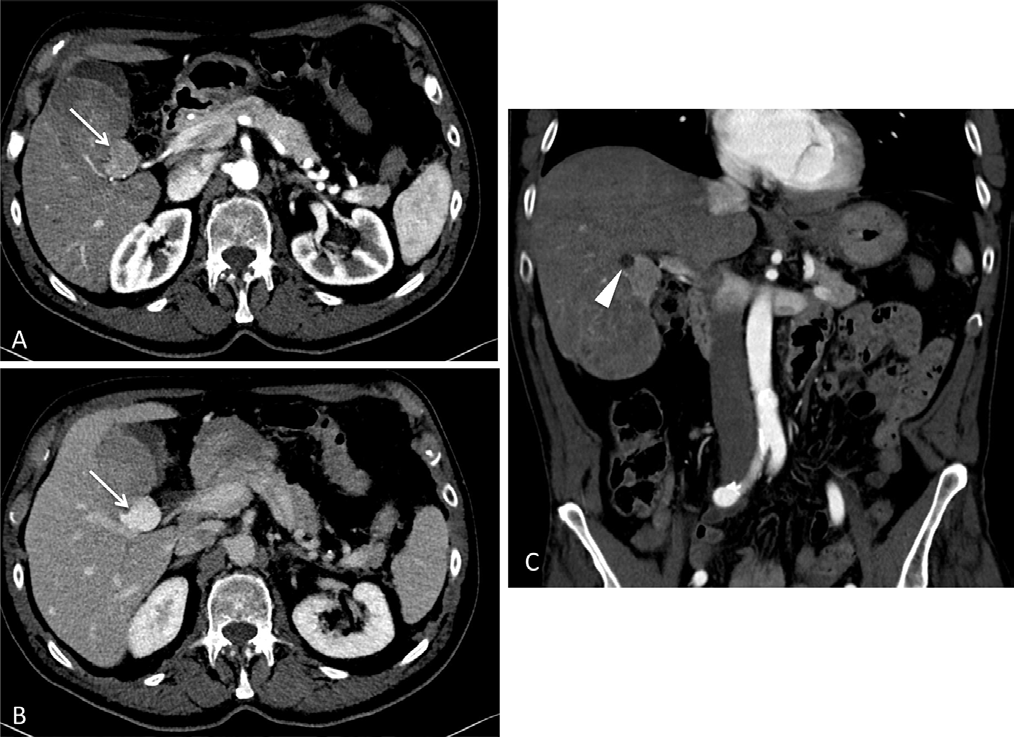
Cranial nodule (white arrow) showed a more conspicuous and homogeneous enhancement respect to the larger lesion (A and B), with a little central cystic area (arrowhead in C).

**Figure 4.**
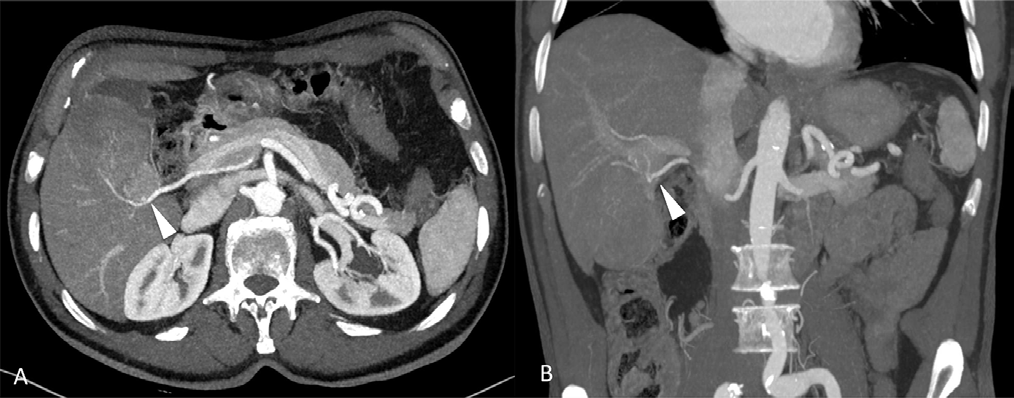
Axial (A) and coronal MIP images (B) show feeding vessels to the lesion arising from the right hepatic artery (arrowhead).

Both the exams showed a large encapsulated lesion, between gallbladder fossa, Vth and IVth liver segments with a peduncolated cranial nodule in the liver hilum. Nonenhanced CT scan showed a hypoattenuating lesion with different areas of marked hypoattenuation (Figure 1). Contrast-enhanced CT scan showed a heterogeneous peripheral enhancement in arterial phase, with slow centripetal enhancement in equilibrium phase (Figure 2), while cranial nodule showed a more conspicuous and homogeneous enhancement, with a little central cystic area (Figure 3). Lesion showed feeding vessels arising from the right hepatic artery (Figure 4). Gallbladder, portal vein and bile ducts were secondly compressed. No lymphadenopathies were detected.

**Figure 5.**
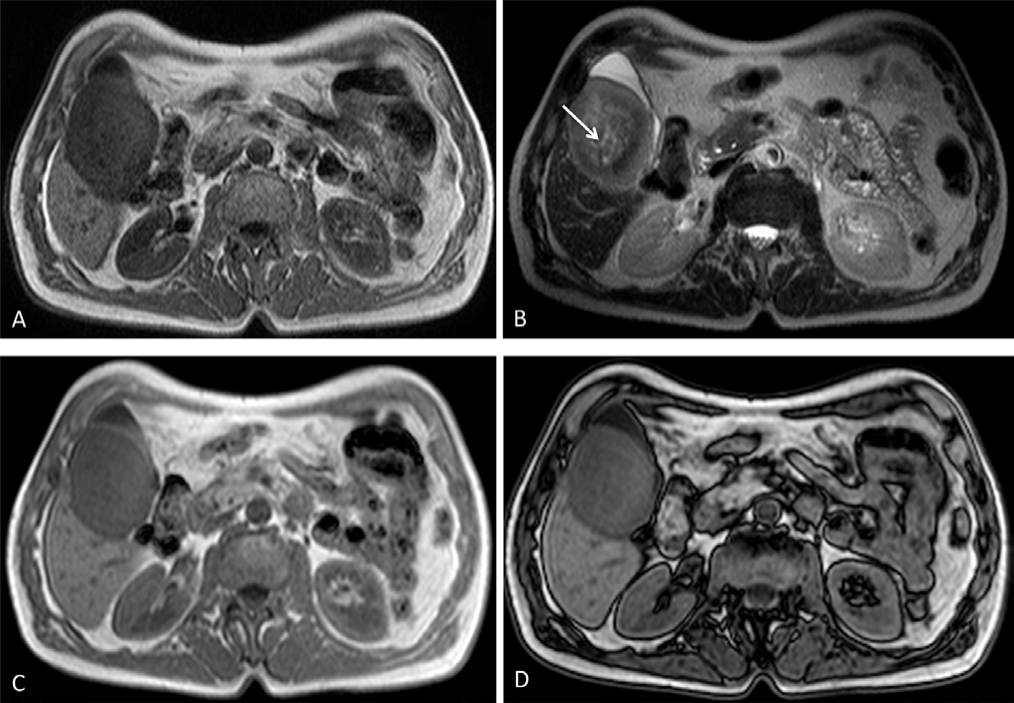
Larger lesion was hypointese in *T*_1_ sequences (A), weakly hyperintense in *T*_2_, with areas of cystic changes (arrow in B), no lipidic areas were detected in the lesion on opposed phase images (C, D). Note lesion between gallbladder and liver parenchyma.

In regard to MRI characteristics (Figure 5), lesion was hypointese in *T*_1_ sequences, weakly hyperintense in *T*_2_, with areas of cystic changes after e.v. contrast administration.

Nodule in the liver hilum was conversely strongly hypointense both in *T*_2_ and *T*_1_ sequences.

No lipidic areas were detected in the lesion on opposed phase and STIR images.

**Figure 6.**
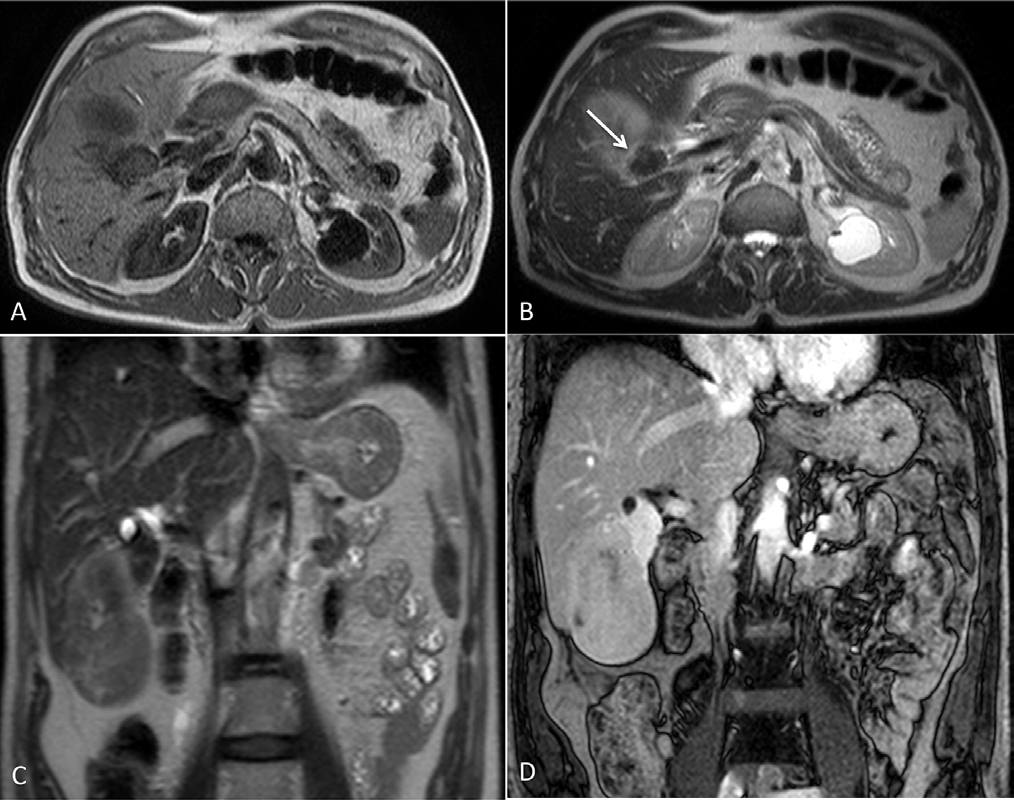
Nodule in the liver hilum was strongly hypointense both in *T*_1_ and *T*_2_ sequences (arrow in A and B). Coronal *T*_2_ (C) and post contrast *T*_1_ (D) image show the double component of the mass with different MRI signal, as a dual-stage lesion.

According to imaging characteristics and laboratory markers radiologist suggested diagnosis of a huge liver encapsulated mesenchimal tumor arising from glisson capsule. Interestingly this lesion showed a “two face” appearance, as the peduncolated nodule in the hilum, seemed to be the same lesion in an earlier stage of evolution, due to a more conspicuous enhancement and homogeneous hypointensity signal respect to the bigger more heterogeneous lesion (Figure 6).

## Treatment

Patient was candidated to surgery and a preoperative evaluation of the future liver remnant volume (FLRV) was performed, using the open-source OsiriX PAC software system with a stand-alone Apple computer. Liver volumes according to Couinaud segmentation were manually performed, and the result showed sufficient remnant liver volume. Surgeons chose a conservative management consisting in the excision of the lonely lesions with a subsegmentectomy approach as they appeared encapsulated at imaging.

**Figure 7.**
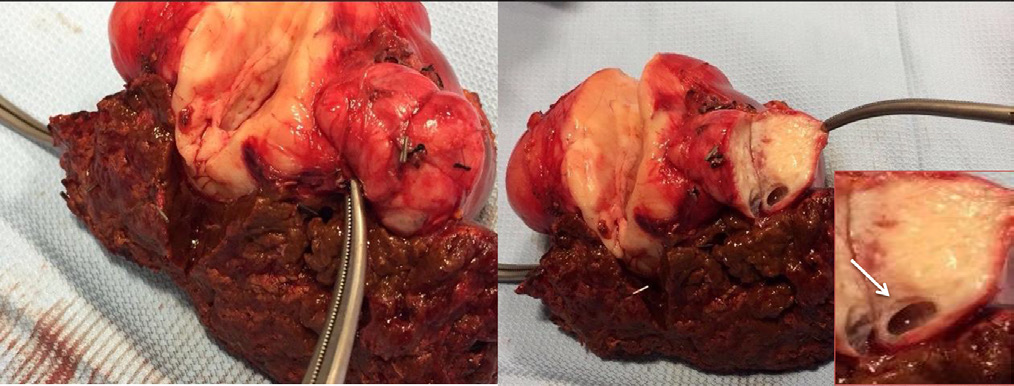
Cut surface of hepatic tissue, near surgical edge. Lesion showed a pattern of growth completely exophitic along liver capsule, it was tender and well capsulated with a peduncolated little nodule; In box the little cystic area within the little nodule (arrow).

At exploratory laparotomy the mass measured 9 × 7 × 4 centimeters, yellow in color, was tender, encapsulated and located next to gallbladder, with a peduncolated nodule in hepatic hilum (Figure 7). No other lesions were found in liver and in abdominal cavity.

Hepatic subsegmentectomy of S5, S4b and cholecystectomy “en bloc” was performed.

**Figure 8.**
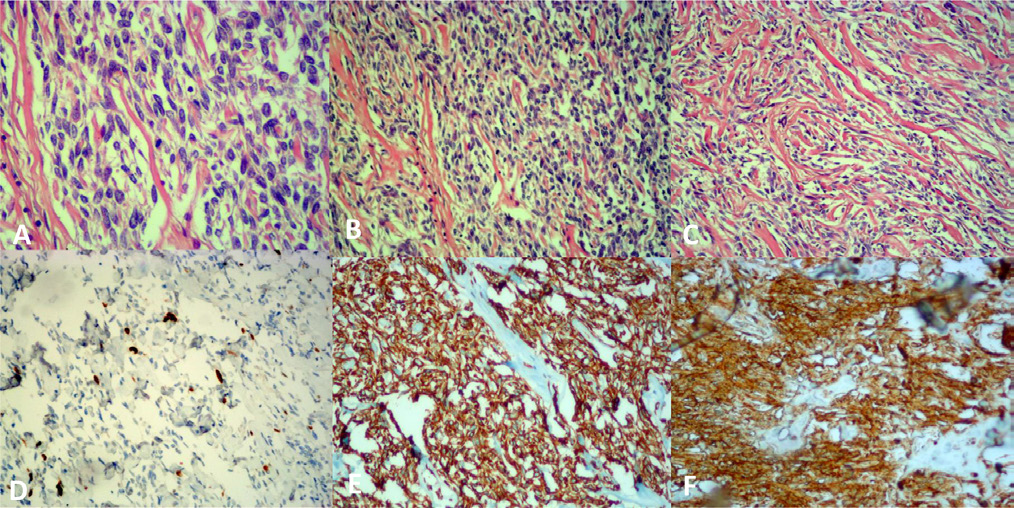
Presence of mitotic figures (A), hematoxylin/eosin staining (B, C): The morphological pattern shows a proliferation, not circumscribed and highly cellulose, consisting of small and medium cellular elements.The cells have a roundish or fused core, a “pattern” of infiltrative growth. The immunocytochemical determination for Ki67 protein shows an intense brownish (nuclear) color in neoplastic cells (D). The immunocytochemical determination for the Vimentin protein shows a diffuse and strong brownish color (cytoplasmic with membrane reinforcement) in the neoplastic cells (E). The immuno hystochemical determination for CD34 protein shows a diffuse and strong brownish (cytoplasmic) staining in neoplastic cells (F).

Microscopically the tumor revealed a high hypercellular proliferation of little and medium cells with round or fusiform nucleus and infiltrative pattern of growth. Immunohistochemical study results showed that cells were strongly positive for vimentin, desmin and CD 34, negative for cytokeratin, LCA, CD 10, CD 31m actin, HMB-45 and S-100 (Figure 8). The pathological diagnosis was malignant fibrous hystiocitoma of the liver. Surgical margins were tumor free.

## Outcome and follow up

The postoperative period was uneventful and the patient was discharged from the hospital 6 days after surgery. After surgery no chemotherapy was administered. The patient is alive and in good health, he received regular follow-up CT examinations at our Institute for 18 months, laboratory tests as AST, ALT and alpha-fetoprotein at the last follow up were 14 (U/L), 16 (U/L) and 6 (ng/ml) respectively.

## Discussion

Malignant fibrous hystiocitoma represents a relatively ”young” pathological entity, as it was first described by Orien and Stout in 1964. It can be considered a sarcomatous lesion usually involving the deep fascia, extremities, or retroperitoneum.^1^ Clinical presentation is variable as patients may present abdominal pain, nausea, vomiting and weight loss, due to the mass effect of these lesions that usually are discovered when they reach a significant volume (mean diameter at diagnosis ranges from 7 to 24 cm).^2^ Liver presentation is very unusual,^3^ generally hepatic MFH is discovered in middle aged patients (50–60 years old) with a slight male predominance and prognosis is very poor, with a 2-year survival rate approximately of 60%, with 20% suffering from local recurrence.^4^ Five histological subtypes have been described: storiform pleomorphic, giant cells, myxoid, inflammatory, and angiomatoid.^5–7^ Laboratory tests may be variable, some patients presented leukocytosis, abnormal liver transaminases and alkaline phosphatase, others may present laboratory results completely negative.^1–4^ This variable clinical presentation and biologic behavior often make accurate diagnosis really challenging. A variable spectrum of imaging appearances have indeed been described, however some characteristic imaging CT and MRI features may suggest this difficult diagnosis. According to different studies^3, 8,9^ liver MFH presents an inhomogeneous low density at precontrast CT, usually with heterogeneous enhancement because of necrotic areas at contrast-enhanced CT and MRI, in some cases a pseudocapsule of delayed enhancement may be depicted. Local recurrence is a common finding and correlates with the depth and size of the primary tumor.^9^ No infiltrative behavior has been described with absence of portal vein invasion, bile duct obstruction or lymph node metastasis.^8^Li et al^10^ described four cases of inflammatory MFH of head and neck showing a similar pattern at imaging, with intense enhancement during arterial phase which persisted in the portal and delayed phases. At MRI primary hepatic MFH show an intermediate low signal on *T_1_* weighted images and heterogeneous high signal intensity on *T*_2_ weighed images.^3^ The imaging findings of our case were partially in accord with the literature, indeed lesion was large and encapsulated, hypoattenuating at precontrast CT with heterogeneous enhancement in arterial phase, and slow centripetal enhancement in equilibrium phase. The cranial peduncolated nodule showed a more conspicuous and homogeneous enhancement, with a little central cystic area. At MRI the larger lesion was hypointese on *T*_1_ sequences, weakly hyperintense on *T*_2_, with areas of cystic changes after e.v. contrast administration whereas the nodule in the liver hilum was strongly hypointense both on *T*_2_ and *T*_1_ sequences. The marked hypointensity of the cranial nodule on *T_2_* weighted images has not yet been described in literature, and probably it is correlated to the high vascular matrix of the pedunculated lesion. Neither signs of infiltration of adjacent structures nor lymphadenopathies were detected. To the best of our knowledge no other cases of liver MFH presenting as completely exophytic mass with a dual stage behavior have been discussed in literature.

## Learning points

1. The present case illustrates CT and MRI findings of a liver primary MFH completely exophytic along liver capsule, imaging allowed the correct attribution of the mass to the liver thanks to the identification of the feeding vessels.
2. Contrast enhancement characteristics as MRI features correctly suggested the mesenchymal nature of the lesion appearing as a dual stage tumor.
3. At imaging the mass showed an expansile behavior, so surgeons chose a conservative subsegmentectomy approach according to CT evaluation of the future liver remnant volume (FLRV).
